# Hippocampal injections of soluble amyloid-beta oligomers alter electroencephalographic activity during wake and slow-wave sleep in rats

**DOI:** 10.1186/s13195-023-01316-4

**Published:** 2023-10-13

**Authors:** Audrey Hector, Chloé Provost, Benoît Delignat-Lavaud, Khadija Bouamira, Chahinez-Anissa Menaouar, Valérie Mongrain, Jonathan Brouillette

**Affiliations:** 1 Department of Pharmacology and Physiology, Université de Montréal, Montréal, Québec Canada; 2 Center for Advanced Research in Sleep Medicine, CIUSSS-NIM, Montréal, Québec Canada; 3 Centre de Recherche, Centre Hospitalier de l’Université de Montréal, Montréal, Québec Canada; 4 Department of Neuroscience, Université de Montréal, Montréal, Québec Canada

**Keywords:** Alzheimer’s disease (AD), Pathological markers, Hippocampus, Sleep architecture, Sleep duration, EEG spectral analysis, Animal model of neurodegeneration

## Abstract

**Background:**

Soluble amyloid-beta oligomers (Aβo) begin to accumulate in the human brain one to two decades before a clinical diagnosis of Alzheimer’s disease (AD). The literature supports that soluble Aβo are implicated in synapse and neuronal losses in the brain regions such as the hippocampus. This region importantly contributes to explicit memory, the first type of memory affected in AD. During AD preclinical and prodromal stages, people are also experiencing wake/sleep alterations such as insomnia (e.g., difficulty initiating sleep, decreased sleep duration), excessive daytime sleepiness, and sleep schedule modifications. In addition, changes in electroencephalographic (EEG) activity during wake and sleep have been reported in AD patients and animal models. However, the specific contribution of Aβo to wake/sleep alterations is poorly understood and was investigated in the present study.

**Methods:**

Chronic hippocampal injections of soluble Aβo were conducted in male rats and combined with EEG recording to determine the progressive impact of Aβ pathology specifically on wake/sleep architecture and EEG activity. Bilateral injections were conducted for 6 consecutive days, and EEG acquisition was done before, during, and after Aβo injections. Immunohistochemistry was used to assess neuron numbers in the hippocampal dentate gyrus (DG).

**Results:**

Aβo injections did not affect the time spent in wakefulness, slow wave sleep (SWS), and paradoxical sleep but altered EEG activity during wake and SWS. More precisely, Aβo increased slow-wave activity (SWA; 0.5–5 Hz) and low-beta activity (16–20 Hz) during wake and decreased theta (5–9 Hz) and alpha (9–12 Hz) activities during SWS. Moreover, the theta activity/SWA ratio during wake and SWS was decreased by Aβo. These effects were significant only after 6 days of Aβo injections and were found with alterations in neuron counts in the DG.

**Conclusions:**

We found multiple modifications of the wake and SWS EEG following Aβo delivery to the hippocampus. These findings expose a specific EEG signature of Aβ pathology and can serve the development of non-invasive and cost-effective markers for the early diagnosis of AD or other amyloid-related diseases.

**Supplementary Information:**

The online version contains supplementary material available at 10.1186/s13195-023-01316-4.

## Background

Alzheimer’s disease (AD) is the most common neurodegenerative disorder among people over 65 years old and affects approximately one in three individuals over 85 years of age [1]. It is established that soluble low-molecular-weight amyloid-beta oligomers (Aβo) resulting mostly from Aβ_1–42_ peptide aggregation are neurotoxic and participate in synapse and neuronal losses [2–6]. Of importance is that Aβo start to accumulate in the brain 10 to 20 years before clinical symptoms of AD [7–11]. Thus, acting early on Aβ pathology (e.g., when cell death begins and restricts to only some brain areas such as the hippocampus) could prevent, or at least slow down, the neurodegenerative process at the onset of AD. Since Aβ pathology was reported to appear before tau pathology, brain atrophy, memory decline, and clinical diagnosis of AD [7, 11], determining the specific signature of soluble Aβo on the brain could also serve to develop required biomarkers to reliably detect AD before neurodegeneration produces irreversible brain damage and profound cognitive deficits.

Sleep disturbances are predominant in AD. For instance, AD patients were reported to have decreased sleep efficiency and total sleep time, with less time spent in both slow-wave sleep (SWS) and paradoxical sleep (PS) [12–15]. Poor sleep quality (e.g., difficulties initiating and maintaining sleep) and less SWS have also been shown in individuals with amnestic mild cognitive impairment (aMCI), a prodromal stage of AD [16, 17]. Furthermore, aMCI patients exhibit alterations in electroencephalographic (EEG) activity during sleep, such as a generally slower activity during PS and lower delta and theta power during stage 2/3 sleep in comparison with age-matched healthy adults [17, 18]. Interestingly, high levels of cerebrospinal fluid Aβ_1–42_, in comparison with lower levels, have been linked to lower sleep efficiency (measured using actigraphy) in cognitively normal individuals [19]. Nevertheless, the specific consequences of Aβ pathology on wakefulness and sleep EEG remain to be defined.

Sleep perturbations were also observed in various AD transgenic rodent models [20, 21]. Indeed, in models with Aβ pathology such as APPswe/PS1dE9 (APdE9), Tg2576, and TgCRND8 mice, alterations in the time spent awake and asleep were reported, together with modifications of EEG activity during wakefulness and sleep [22–25]. For some of these models, wake/sleep alterations are found before substantial Aβ pathology [22, 24]. It should be noted that Aβ pathology in these models is associated with the development of amyloid plaques, which prevents understanding the specific contribution of Aβo to sleep alterations. Moreover, these transgenic mice overexpress amyloid precursor protein fragments other than Aβ that could impact sleep phenotypes independently of Aβo [26, 27]. Therefore, the specific contribution of soluble Aβo to wake/sleep alterations can be assessed using repeated Aβo injections in the hippocampus [6, 28]. This strategy also allows for the precise targeting of the hippocampus, whose activity during PS was causally linked to memory consolidation [29].

Thus, we performed hippocampal injections of soluble Aβ_1–42_ oligomers in rats and combined this treatment with EEG measurements to define the precise impact of soluble Aβo on wake/sleep architecture and quality. Injections were repeated for 6 consecutive days allowing the assessment of wake/sleep phenotypes in the course of Aβ pathology development/progression. Although wake/sleep architecture was not affected by this treatment, the EEG spectral profile of the frontal cortex was modified, in particular, for wakefulness and SWS. This was associated with a decrease in the ratio between theta (5–9 Hz) activity and slow-wave (0.5–5 Hz) activity (SWA) in both states and was significant after 6 days of Aβo injections but not after 2 or 5 days. These findings suggest that the specific signature of soluble Aβo-induced neurodegeneration on wake/sleep EEG features could be used as an early biomarker of AD, when soluble Aβo accumulation occurs and other pathological, structural, and clinical events related to AD are undetectable.

## Methods

### Animals and protocol

Twenty Long-Evans male rats (Charles River, Kingston, NY, USA) were used and housed individually in a 12-h light/12-h dark cycle with ad libitum access to food and water. Rats were 3 months old and weighed approximately 300–400 g at the time of surgery for implantation of intra-hippocampal cannulas, EEG, and electromyographic (EMG) electrodes. Animals were randomly assigned to two experimental groups: a control group receiving hippocampal injections of amyloid-beta scrambled (Aβscr; *n* = 11) and a group receiving soluble Aβo for 6 consecutive days (*n* = 9). Six consecutive days of injection was selected to benefit from a well-characterized model [6, 28, 30], together with targeting the early and short-term impacts of Aβo, which is relevant to the identification of wake/sleep phenotypes related to the onset of Aβ pathology and prodromal stages of AD. EEG/EMG recordings were done before, during, and after 6 consecutive days of intra-hippocampal injections, and rats were sacrificed by decapitation 14 days after the last injection. The brains were rapidly sampled, frozen on dry ice, and stored at − 80 °C until processing. The adequate positioning of the cannulas in the hippocampus was confirmed using Nissl staining before EEG analyses, and neuronal cell count in the dentate gyrus (DG) was done using immunohistochemistry (IHC). Four additional rats were submitted to intra-hippocampal injections for 6 consecutive days without EEG/EMG electrode implantation (Aβscr, *n* = 2; Aβo, *n* = 2), and their brain was sampled 24 h after the last injection to verify the distribution of Aβ in the hippocampus at the time corresponding to the post-injection EEG/EMG recording of the previously described animal cohort. All manipulations were conducted according to the guidelines of the Canadian Council on Animal Care and approved by the *Comité d’éthique de l’expérimentation animale* of the CIUSSS-NIM.

### Cannula and electrode implantation surgery

**Fig. 1:**
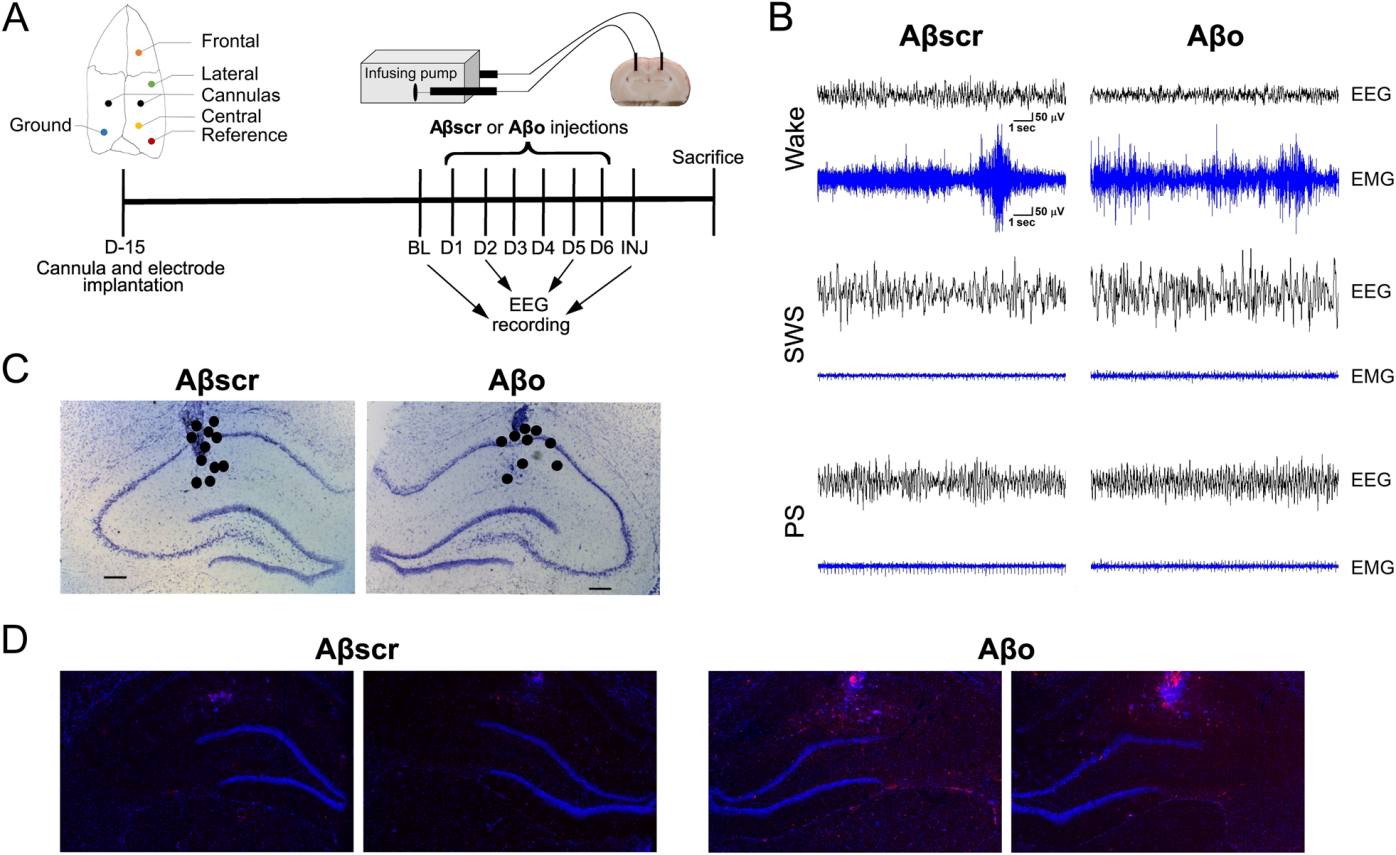
Experimental protocol, representative EEG and EMG traces, and validation of the position of implanted cannulas and of Aβ spreading. **A** Schematic representation of the position of cannulas (in CA1) and electrodes (surface of the cerebral cortex) and timeline of the experimental protocol. Ten-min infusions of 2-µL Aβscr or Aβo were performed every morning for 6 days. The EEG signal was recorded at baseline (BL) 24 h before beginning the injections, on days 2 (D2) and 5 (D5) of the injections, and 24 h following the last injection (INJ). **B** Representative EEG and EMG traces of the frontal-central bipolar signal obtained for wakefulness, SWS, and PS during the INJ day in one rat submitted to Aβscr injections and one to Aβo injections. **C** Approximate position of the cannulas in the hippocampus was determined using Nissl staining and marked with black dots for Aβscr (*n* = 11) and Aβo (*n* = 9) animals. Scale bars = 300 μm. **D** IHC images (magnification × 2.5) of Aβ staining (clone 6E10; red) in the hippocampus shown with DAPI staining (blue) in four rats submitted to 6 days of Aβscr (left) or Aβo (right) injections but not to EEG/EMG instrumentation. The position of the cannula tip is also observable for each rat

Two weeks after arrival, rats were implanted with bilateral cannulas (3260PG/SPC; Plastic One/Protech International Inc., Boerne, TX, USA; cut at 0.36 cm under the pedestal) and EEG/EMG electrodes (Fig. 1A). Rats were anesthetized (70 mg/kg ketamine and 10 mg/kg xylazine, i.p.; together with constant inhalation of isoflurane 0.5–2.5%) and installed in a stereotaxic frame (David Kopf Instrument, CA, USA). Cannulas were implanted in the dorsal hippocampus (coordinates relative to bregma: - 0.36 cm anteroposterior (AP), ± 0.22 cm mediolateral (ML) [31]) with the pedestal placed at the level of the skull surface. Three EEG electrodes, a reference electrode, and a ground (all miniature screws model FF00CE125; J.I. Morris Inc., Southbridge, MA, USA) were implanted at the surface of the cerebral cortex during the same surgical procedure (relative to bregma: frontal electrode AP + 0.3 cm, ML - 0.1 cm; lateral electrode AP - 0.07 cm, ML - 0.3 cm; central electrode AP - 0.61 cm, ML - 0.1 cm; reference electrode AP - 0.81 cm, ML - 0.40 cm; see Fig. 1A) followed by implantation of two EMG electrodes in the neck muscles similar to previously performed [32]. Cannulas and electrodes were fixed on the skull surface with dental cement, and the skin was sutured using PERMA-HAND® silk suture 4–0 (Ethicon, Johnson & Johnson, Markham, ON, Canada). Body temperature was monitored during surgery, and kept at 37 ± 0.1 °C using a heat pad. Rats were allowed to recover for 2 weeks following surgery before starting Aβo intra-hippocampal injections. The surgery for the four additional rats was the same except that no electrode was implanted.

### Aβ preparation and injection

Solutions of Aβo and Aβscr were prepared as described previously [6, 28]. Briefly, Aβ_1–42_ (rPeptide, GA, USA) and Aβscr_1–42_ peptides (rPeptide) were dissolved at a concentration of 1 mg/mL in 99% hexafluoroisopropanol (HFIP) (Sigma-Aldrich, Saint Louis, MO, USA). The HFIP was evaporated using a gentle stream of nitrogen gas, and the peptide film was dissolved at a final concentration of 1 mg/mL in dimethylsulfoxide (DMSO; Sigma-Aldrich). Complete removal of DMSO was achieved by eluting Aβ peptides from a 5-mL HiTrap desalting column (GE Healthcare, Mississauga, ON, Canada) with 50 mM Tris and 1 mM EDTA buffer at pH 7.5. Aβ_1–42_ concentration was measured using a Pierce BCA protein assay kit (Thermo Fisher Scientific, Ottawa, ON, Canada).

To induce the formation of soluble low-molecular-weight Aβo, the Aβ solution was kept at room temperature for 1 h before starting the injection and then kept on ice with a maximum lag time of 30 min before use. The biophysical and biological properties of this Aβ preparation have been previously characterized by others [33, 34]. Starting 2 weeks after surgery, every morning for 6 days (during hours 2 to 6 after light onset), awake and freely moving rats were injected with 2 µL of 0.2 µg/µL soluble Aβo or 0.2 µg/µL Aβscr at a rate of 0.2 µL/min via cannulas and PE50 tubing (Plastics One) connected to a 10-µL Hamilton syringe pump system (KDS310; KD Scientific, Holliston, MA, USA) (Fig. 1A). The tubing was left in place for another 3 min at the end of each injection, and the cannula capped to prevent reflux of the injected solution. The injection concentration was chosen to match previous studies describing this model because it was shown to trigger an accumulation of Aβo and neuronal damage in the DG of the hippocampus [6, 28, 35].

### EEG recording and analyses

Prior to baseline (BL) EEG recordings, animals were habituated to cabling conditions for 72 h. EEG/EMG recordings were performed at BL (24 h before the first injection day), on days 2 (D2) and 5 (D5) of injections, and 24 h following the last intra-hippocampal injection (INJ) (Fig. 1A). BL and INJ recordings started at light onset (Zeitgeber time 0) and were done for 24 h. D2 and D5 recordings lasted 20–21 h since signals were not recorded during the time devoted to injections (3 to 4 h of uncabling per animal). The EEG and EMG signals were amplified (Lamont amplifier), sampled between 256 and 1024 Hz, and filtered using the commercial software Harmonie (Natus, Middleton, WI, USA) as previously performed [36]. Vigilance states (wakefulness, SWS, and PS) were visually assigned to 4-s epoch using a bipolar signal between the frontal and central electrodes as done previously [36–38]. Briefly, wakefulness was identified when low-amplitude/fast-frequency EEG activity was observed together with high-amplitude EMG, SWS when high-amplitude/low-frequency EEG activity was observed with low-amplitude EMG, and PS when regular theta activity was observed for the EEG together with minimal EMG amplitude. The EEG and EMG signals remained of sufficient quality to identify vigilance states after the 6 days of intra-hippocampal injections (Fig. 1B). Artifacts were simultaneously identified and subsequently excluded from spectral analysis. Percent time spent in each state was averaged per 24 h and per h. Spectral analysis was performed using fast Fourier transform to calculate the EEG power density between 0.5 and 55 Hz (0.5-Hz bin resolution) for the unipolar EEG signals of the central and frontal electrodes (signal of sufficient quality for spectral analysis for both central and frontal electrodes in 9 Aβscr and 6 Aβo rats; lateral electrode not usable due to artefactual signal in 4 additional rats). This was done separately for wakefulness, SWS, and PS. Power spectra were computed for BL, D2, D5, and INJ and expressed as a percentage of the 24-h mean of the activity in all bins of all states for each rat. Relative spectral activity was then averaged for six frequency bands for statistical comparisons (i.e., SWA, 0.5–5 Hz; theta, 5–9 Hz; alpha, 9–12 Hz; sigma, 12–16 Hz; beta1, 16–20 Hz; beta2, 20–30 Hz; and gamma, 30–55 Hz). For D5, one additional animal per group was lost due to recording issues (Aβscr, *n* = 8; Aβo, *n* = 5). The time course of SWA, theta, and alpha activities during SWS was also computed for BL and INJ using 12 intervals during the light periods for which an equal number of SWS epoch contributed and 6 equal intervals during the dark period as done previously [36, 38, 39]. Time courses were analyzed using activity per interval expressed relative to the 24-h mean activity for each animal.

### Histology and immunostaining

Ten-micrometer coronal slices were cut at - 20 °C using a cryostat (HM525 NX with blade S35 – Feather®; Thermo Fisher Scientific). Slices were mounted on Superfrost Plus slides (12-550-15; Thermo Fisher Scientific) and placed at - 80 °C until use. To verify the position of implanted cannulas, slides were placed in a 10% formalin solution (HT501128; Sigma-Aldrich), washed with PBS (9808S; Cell Signaling Technology, Danvers, MA, USA), and immersed for 20 min in Nissl staining (cresyl violet acetate 0.5% [Alfa-Aesar portfolio]; Thermo Fisher Scientific). Slides were then successively immersed in 70%, 95%, and 100% ethanol baths. The cannula position was confirmed for the 11 Aβscr rats and the 9 Aβo rats (Fig. 1C).

IHC with NeuN antibody was performed for neuronal cell counting. Slides were fixed for 15 min in 10% formalin, washed with PBS, immersed in a blocking solution (PBS with 5% goat serum [5425; Cell Signaling Technology] and 0.3% Triton X-100 [BP151-500; Thermo Fisher Scientific]) for 60 min at room temperature, and incubated overnight at 4 °C with anti-NeuN antibodies (24307; Cell Signaling Technology) 1:1000 diluted in PBS with 1% BSA (BP1600-100; Thermo Fisher Scientific) and 0.3% Triton X-100. The slides were washed and incubated with a secondary antibody (1:1000 Alexa fluor 488; 4340, Cell Signaling Technology) for 90 min at room temperature and protected from light. The slides were then washed and dried, and one drop of Prolong Gold Antifade DAPI (8961S; Cell Signaling Technology) was applied on each section before being covered with a coverslip (102455; Thermo Fisher Scientific). IHC was done similarly for the brain slices of rats not implanted with electrodes using anti-β-amyloid 1–16 antibodies (1:1000; clone 6E10 Covance catalog SIG-39320; BioLegend, San Diego, CA, USA) followed by secondary antibodies (1:500 Alexa fluor 555; 4409, Cell Signaling Technology). This staining showed that, following six consecutive days of intra-hippocampal Aβo injections, Aβ peptides distribute around the injection site within the hippocampus, reaching the DG (Fig. 1D). No observable staining was found in Aβscr-injected animals.

### Microscopy and cell counting

A Zeiss AxioImager 2 fluorescence microscope, a monochrome camera, and the StereoInvestigator software (MBF Bioscience, Williston, VT, USA) were used to acquire immunofluorescent images. Wavelength filters of 488 nm and 350 nm were used to excite fluorochromes of the secondary antibody and DAPI, respectively. Cell counting was done on images acquired using × 20 magnification. Three regions of interest (ROIs) of the same size per slice were used for NeuN^+^ cell counting, and 3 brain slices were manually counted per animal using the ImageJ software. Some animals (Aβscr, *n* = 3; Aβo, *n* = 5) were excluded due to tissue damage in the targeted area of the DG. Cell counts were compared between the groups using the 3 slices of each animal as different data points and also using the mean of all slices for each animal.

### Statistical analyses

The data are presented as mean ± SEM. Prism 7 (GraphPad Software Inc., La Jolla, CA, USA) was used to prepare the figures and perform the statistical analyses. Two-way or three-way analyses of variance (ANOVAs) were used for the comparison of percent time spent awake and asleep with factors treatment (Aβscr vs Aβo), day (BL vs INJ), and light–dark period. Three-way repeated measure ANOVAs were used to compare the distribution of percent time in states and the time course of activity in frequency bands with factors treatment, day, and h or interval. When appropriate, repeated measure effects were adjusted using the Greenhouse–Geisser correction. Paired *t*-tests were used to compare the activity in different frequency bands between BL and INJ for each group separately and activity ratios, and unpaired *t*-test to compare DG cell counting. One-way repeated measure ANOVAs were used to compare the activity in different frequency bands between BL, D2, and D5. The results of statistical comparisons of the activity in the different frequency bands are presented in Additional file 1: Tables S1 and S2. The threshold for statistical significance was set at 0.05, and significance levels are identified with *p < 0.05 and **p < 0.01.

## Results

### Wake/sleep amount is preserved after Aβo injections

**Fig. 2:**
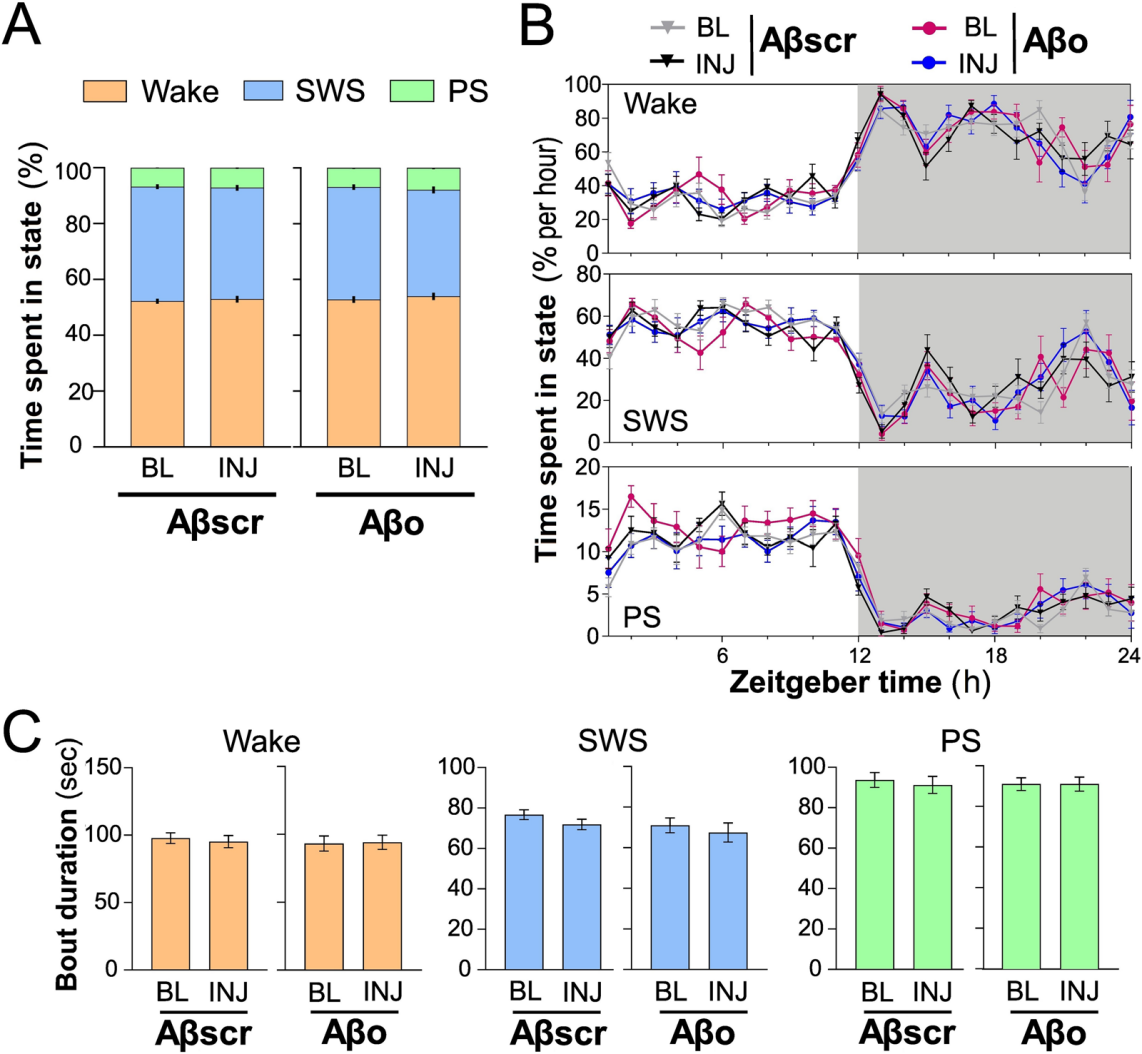
Wake/sleep architecture variables in Aβscr- and Aβo-injected rats during BL and INJ days. **A** Percent time spent in wake, SWS, and PS during 24 h at BL and INJ in the two groups. **B** Percent time spent in wake, SWS, and PS computed per h for BL and INJ in each group. **C** Twenty-four-h mean duration of individual bouts of wake, SWS, and PS for BL and INJ in each group. Aβscr, *n* = 11; Aβo, *n* = 9

We first compared the proportion of time spent in each vigilance state (wake, SWS, and PS) during the full 24 h at BL and the full 24 h after the last injection (INJ). The percent time spent in each state did not significantly differ between Aβscr and Aβo-injected rats (Fig. 2A; for wake, treatment by day interaction *F*_1,18_ = 0.04, *p* = 0.8, treatment effect *F*_1,18_ = 0.4, *p* = 0.5; for SWS, treatment by day interaction *F*_1,18_ = 0.3, *p* = 0.6, treatment effect *F*_1,18_ = 1.1, *p* = 0.3; for PS, treatment by day interaction *F*_1,18_ = 1.5, *p* = 0.2, treatment effect *F*_1,18_ = 1.2, *p* = 0.3). Similarly, Aβo did not impact the percent time spent in each vigilance state during the 12-h light and 12-h dark periods (Additional file 1: Fig. S1A; treatment by day by light–dark interactions *F*_1,18_ < 1.6, *p* > 0.2; treatment by day interactions *F*_1,18_ < 1.6, *p* > 0.2; treatment by light–dark interactions *F*_1,18_ < 0.3, *p* > 0.6; treatment effects *F*_1,18_ < 1.2, *p* > 0.2). When analyzing the time spent in vigilance state over the 24 h, we observed a normal distribution of wakefulness and sleep states for both groups, with less time in wake and more in sleep during the light period, and more time in wake and less in sleep during the dark period (Fig. 2B; h effects *F*_23,184/230_ > 12.6, *p* < 0.0001). There was no significant difference in the distribution of time spent awake and asleep between BL and INJ for both Aβscr- and Aβo-injected rats (day by h interactions *F*_23,184/230_ < 2.2, *p* > 0.05). In sum, wake/sleep amount and distribution were not altered by 6 days of Aβo administration to the hippocampus.

### Sleep fragmentation/consolidation is unaltered by Aβo

Fragmented sleep is characterized by shorter and more frequent individual bouts of states, whereas consolidated wake/sleep is associated with longer and less frequent bouts. Sleep fragmentation has been reported in AD patients and animal models [40–42]. Six days of Aβo injection did not significantly modify the 24-h mean duration of individual bouts of wake, SWS, and PS in comparison with BL (Fig. 2C; for wake, treatment by day interaction *F*_1,18_ = 0.15, *p* = 0.7, treatment effect *F*_1,18_ = 0.3, *p* = 0.4; for SWS treatment by day interaction *F*_1,18_ = 0.14, *p* = 0.7, treatment effect *F*_1,18_ = 1.3, *p* = 0.3; for PS treatment by day interaction *F*_1,18_ = 0.17, *p* = 0.6, treatment effect *F*_1,18_ = 0.1, *p* = 0.7). The number of long and short episodes of states during BL and INJ days was also compared between the groups to further assess fragmentation/consolidation. The number of short wake and SWS episodes (4-s episodes) did not significantly differ between the groups and days (Additional file 1: Fig. S1B; treatment by day interactions *F*_1,18_ < 0.8, *p* > 0.4; treatment effects *F*_1,18_ < 1.9, *p* > 0.2). The number of long wake episodes (8 and 16 min) and long SWS and PS episodes (60 s) was also unaltered by Aβo after 6 days of hippocampal injections (Additional file 1: Fig. S1C; treatment by day interaction *F*_1,18_ < 1.3, *p* > 0.2; treatment effects *F*_1,18_ < 2.3, *p* > 0.1). Therefore, wake/sleep fragmentation/consolidation was globally preserved following the Aβo treatment.

### Altered EEG activity after Aβo injections

**Fig. 3:**
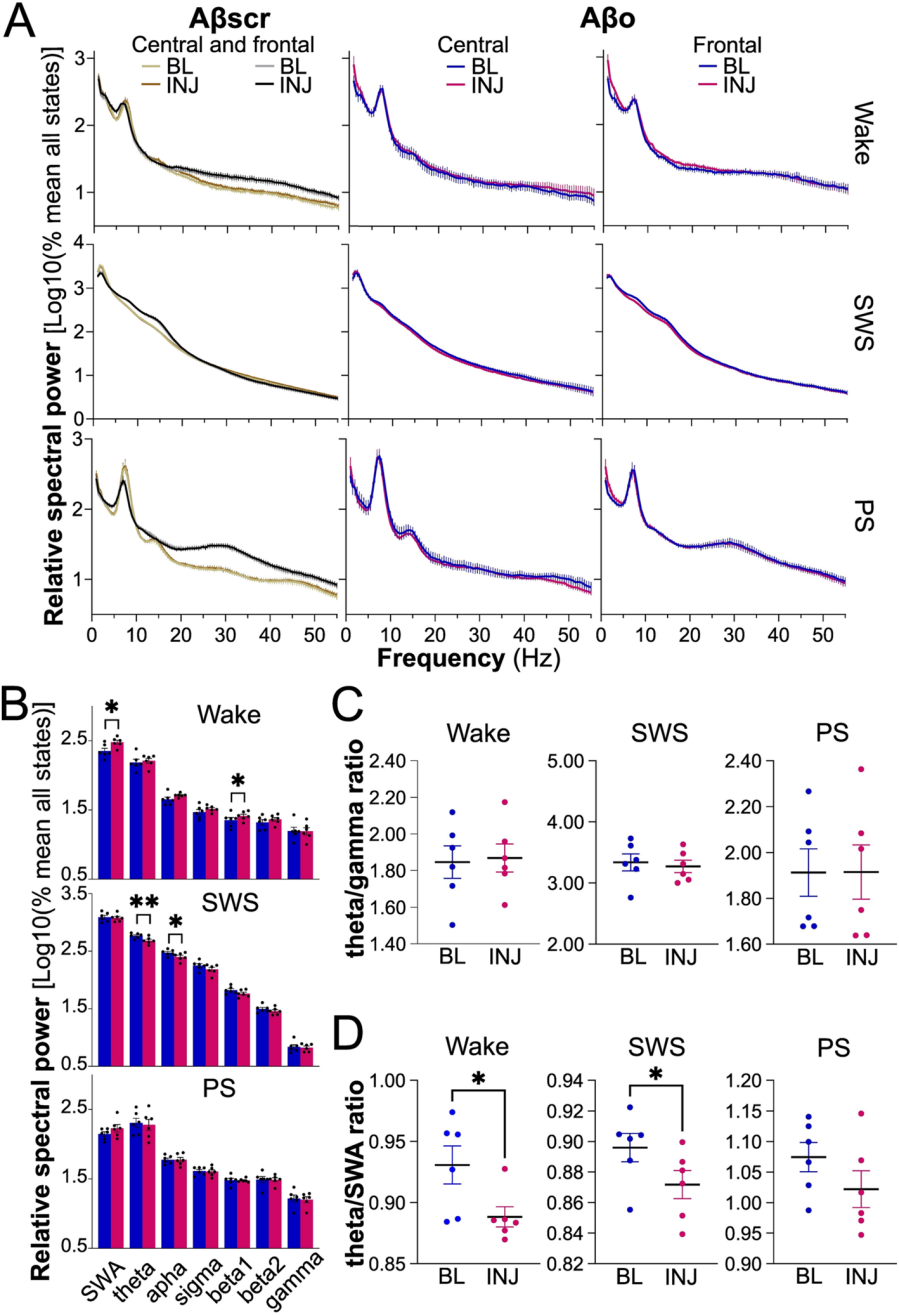
Mean 24-h spectral power and activity ratios for the frontal and central electrodes during BL and INJ days in Aβo and Aβscr groups. **A** Twenty-four-h spectral power of the central and frontal electrodes during wake, SWS, and PS in Aβscr- and Aβo-injected rats at BL and INJ. **B** Spectral activity in each frequency band (SWA, 0.5–5 Hz; theta, 5–9 Hz; alpha, 9–12 Hz; sigma, 12–16 Hz; beta1, 16–20 Hz; beta2, 20–30 Hz; gamma, 30–55 Hz) during wake, SWS, and PS for Aβo-injected rats at BL (blue) and INJ (pink) for the frontal electrode (*p < 0.05; **p < 0.01, BL vs INJ). **C** Ratio of theta activity on gamma activity for the Aβo group at BL and INJ for the frontal electrode. **D** Ratio of theta activity on SWA for the Aβo group at BL and INJ for the frontal electrode (*p < 0.05, BL vs INJ). Aβscr, *n* = 9; Aβo, *n* = 6

Spectral analysis of the EEG was performed to verify whether soluble Aβo impact the quality of wakefulness and sleep states (Fig. 3). This was done for the frontal and central electrodes to evaluate the effects of Aβo on a region of the cerebral cortex more distal to the injection sites (frontal) and on a region capturing better hippocampal activity (central). For both electrodes, 24-h mean spectral power between 0.5 and 55 Hz was indistinguishable between BL and INJ for the three vigilance states in the Aβscr control group (Fig. 3A first column, Additional file 1: Fig. S2A first two columns, and Additional file 1: Table S1). In the Aβo group, spectral power during wake, SWS, and PS were also not significantly different between BL and INJ for the central electrode (Fig. 3A second column, Additional file 1: Fig. S2A last column, and Additional file 1: Table S1). In contrast, Aβo injections changed spectral activity during wake and SWS for the frontal electrode (Fig. 3A last column, Fig. 3B, and Additional file 1: Table S1). More precisely, wake SWA and beta1 activity were increased after 6 days of Aβo delivery to the hippocampus, while SWS theta and alpha activities were decreased.

Given that the relationship between theta and gamma activities was found to be altered in a transgenic mouse model of AD [43], we compared the theta/gamma activity ratio between BL and INJ separately for wake, SWS, and PS. For the Aβscr control group, BL and INJ did not significantly differ in this ratio for both the frontal and central signals (Additional file 1: Fig. S2B; *t* < 1.5, *p* > 0.1). The same was found in Aβo-injected rats (Fig. 3C and Additional file 1: Fig. S2B; *t* < 0.8, *p* > 0.3). The ratio between theta activity and SWA was also analyzed because it is considered a marker of general slowing of the EEG signal and relevant to AD [44–46]. Again, there was no significant difference between BL and INJ in the Aβscr-treated group for both cortical sites and all three states, which also applies to the central electrode in the Aβo group (Additional file 1: Fig. S2C; *t* < 1.7, *p* > 0.1). However, 6 days of Aβo injection significantly decreased the theta activity/SWA ratio computed during wakefulness and SWS when considering the frontal electrode (Fig. 3D; for wake *t* = 3.2, *p* = 0.02; for SWS *t* = 3.7, *p* = 0.01; and for PS *t* = 2.2, *p* = 0.07). Overall, when considering the average activity over the full nychthemeron, the administration of Aβo to the hippocampus during 6 consecutive days was found to impact EEG activity and to modify the relationship between the activity in slower frequency bands, specifically during wakefulness and SWS, and only for the frontal cortical site.

**Fig. 4:**
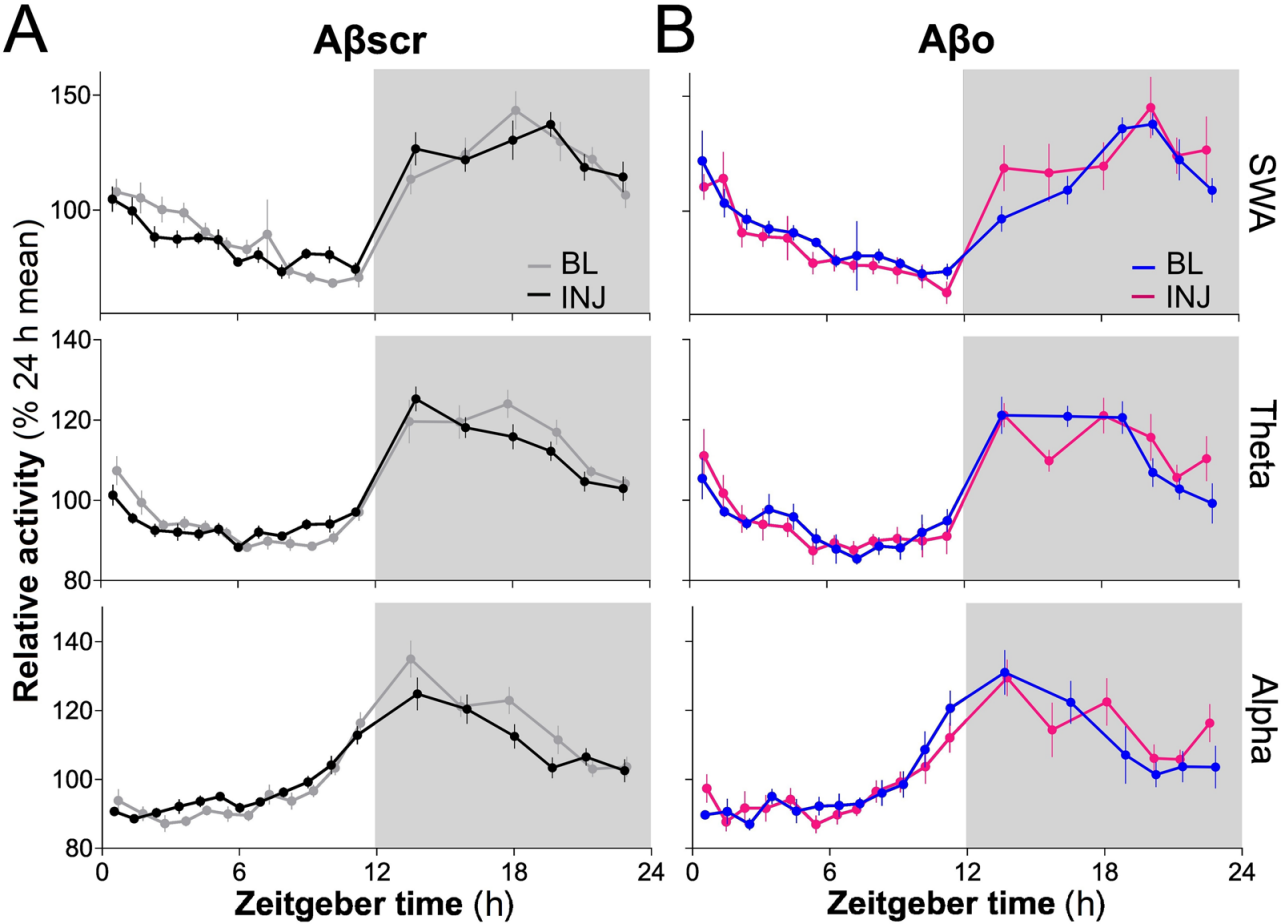
Time course of SWA, theta, and alpha activity during SWS for BL and INJ days in Aβscr- and Aβo-injected rats. **A** The 24-h variations at the frontal electrode of SWA (0.5–5 Hz), theta (5–9 Hz), and alpha (9–12 Hz) activity in the Aβscr group (*n* = 9). **B** The 24-h variations at the frontal electrode in the same frequency bands in Aβo-injected rats (*n* = 6)

To further interrogate the impact of hippocampal Aβo on the regulation of EEG activity, we examined the 24-h dynamics of SWA, theta, and alpha activities during SWS for the frontal electrode. SWA dynamics was interrogated given its recognized relationship with homeostatic sleep regulation [47–50], while theta and alpha bands were included to assess whether changes in the mean 24-h activity (Fig. 3B) are associated with changes in daily variations. In the Aβscr group, the time course of activity in the targeted frequency bands did not significantly differ between BL and INJ (Fig. 4A; day by interval interactions *F*_17,272_ < 1.6, *p* > 0.08; day effects *F*_1,16_ < 1.0, *p* > 0.3). Similar observations were made in the Aβo group (Fig. 4B; day by interval interactions *F*_17,170_ < 1.1, *p* > 0.3; day effects *F*_1,10_ < 2.1, *p* > 0.1). Altogether, these results indicate that the daily dynamics of SWS SWA, theta, and alpha activities is preserved after hippocampal Aβo injections.

### Six days of injection are necessary to alter EEG activity

**Fig. 5:**
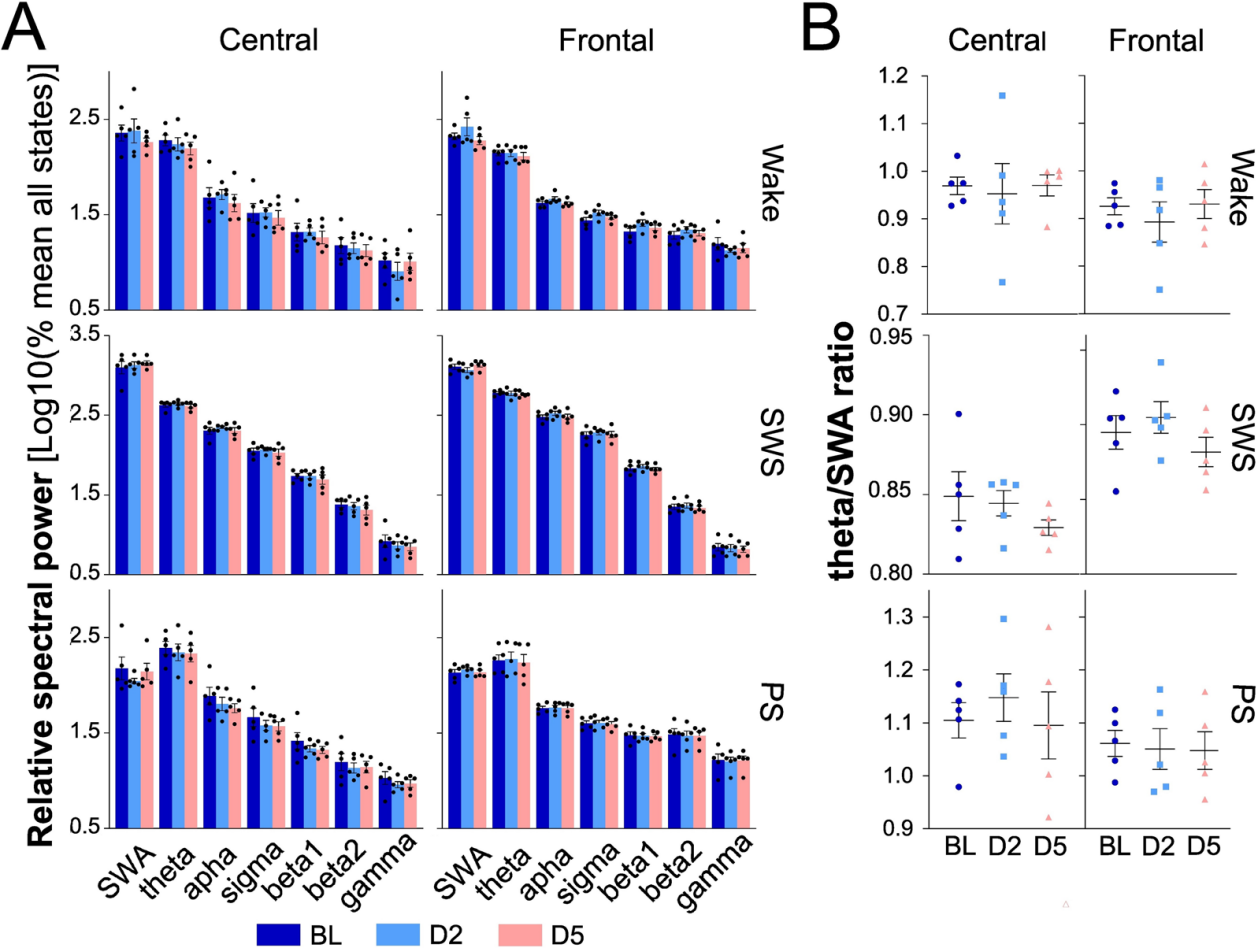
Wake, SWS, and PS spectral activity in each frequency band and theta activity/SWA ratio at BL, D2, and D5 in Aβo-injected rats (*n* = 5). **A** SWA, theta, alpha, sigma, beta1, beta2, and gamma activity averaged over the recording period for the central and frontal electrodes. **B** Ratio of theta activity on SWA for the central and frontal electrodes averaged over the recording period in the Aβo group

To determine whether EEG alterations are detectable before 6 days of Aβo injections, EEG spectral activity in the different frequency bands was compared between BL, D2, and D5 in the Aβscr and Aβo groups. As expected, wake, SWS, and PS spectral activities at the frontal and central electrodes did not significantly differ at D2 and D5 in comparison with BL in the Aβscr group (Additional file 1: Fig. S3, and Table S2). Similar observations were made for the Aβo group (Fig. 5A, Additional file 1: Table S2). We also examined the theta activity/SWA ratio in Aβo-injected rats and found no significant change at D2 and D5 in comparison with BL for both electrodes and all states (Fig. 5B; frontal electrode *F*_2,12_ < 1.3, *p* > 0.3; central electrode *F*_2,12_ < 1.0, *p* > 0.3). Thus, these results suggest that a minimum of 6 days of hippocampal Aβo injections is required to detect alterations in wakefulness and SWS EEG activity.

### Decrease DG neuronal count after Aβo injections

**Fig. 6:**
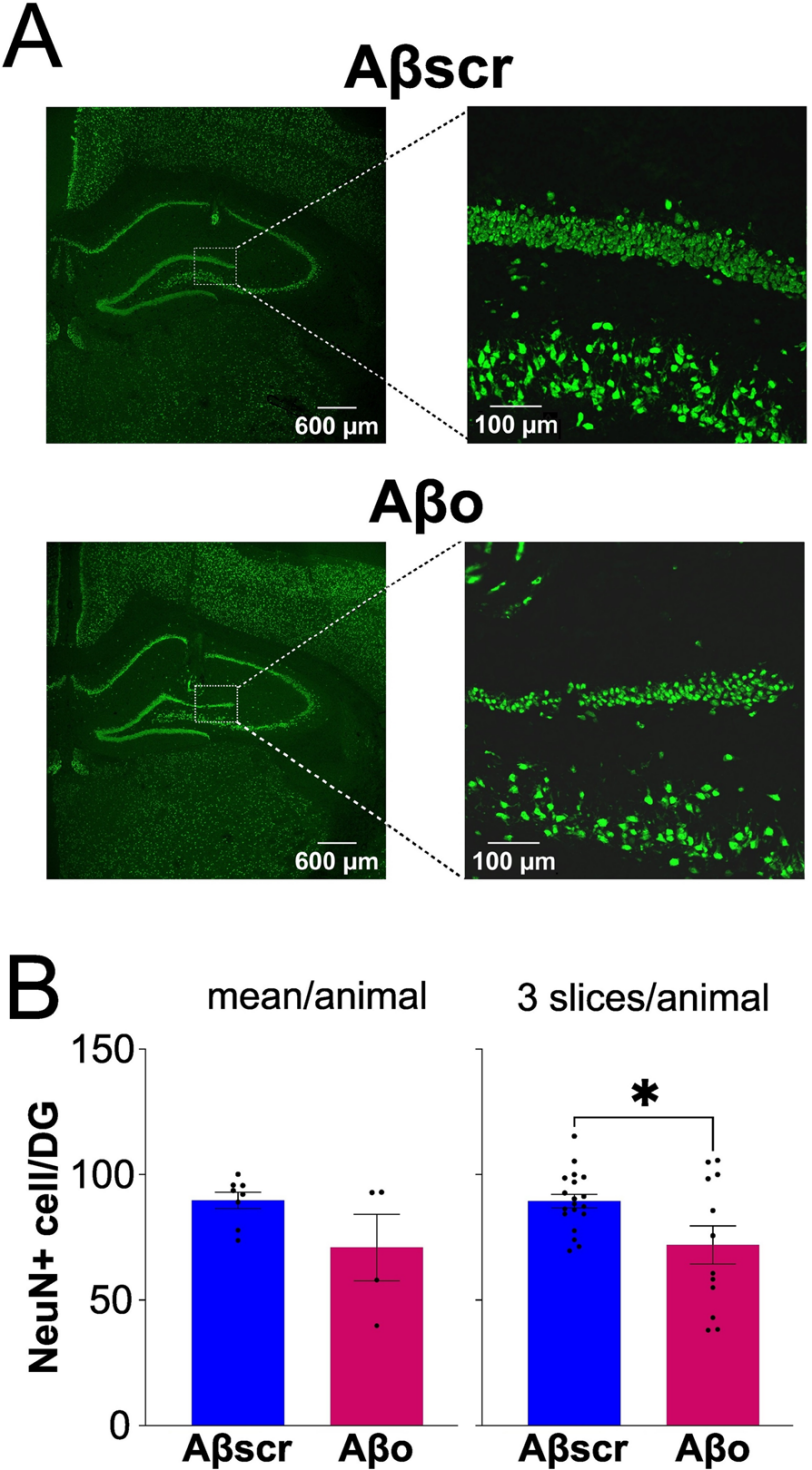
Dentate gyrus (DG) neuronal count of the granular cell layer in the Aβscr (*n* = 8) and Aβo (*n* = 4) groups. **A** Representative IHC images of NeuN staining (green) in the full hippocampus (magnification × 2.5) showing the approximate location where neuron counting was done (white rectangle, and magnification × 20). **B** Number of NeuN-positive cells in the granular cell layer compared between the groups using two different calculations: on the left, the mean of three slices per animal; on the right, three slices for each animal used as different data points (*p < 0.05, Aβscr vs Aβo)

Post-mortem analysis of DG neuronal integrity was conducted in the two groups, given that the DG is located directly under the injection site and not physically damaged by cannulation in comparison with area CA1 (Fig. 6A). When considering each animal as a biological replicate for analysis, the number of NeuN-positive cells showed a tendency to be lower in the Aβo group compared to the Aβscr group (Fig. 6B; *t* = 1.9, *p* = 0.09). Given the relatively small number of animals available for quantification, the mean per slice (3 slices per animal) was also compared and revealed a significantly lower number of NeuN-positive cells in the Aβo group compared to Aβscr animals (*t* = 2.5, *p* = 0.02). This result is in line with observations in previously published work [6, 51] that repeated injections of soluble Aβo lead to a noticeable loss of hippocampal neurons.

## Discussion

Our results indicate that hippocampal soluble Aβo alone drive various alterations in wakefulness and sleep quality (as indexed by EEG activity) without any change in wake/sleep amount and alternation. By performing chronic injections over 6 days in male rats, we observed that soluble Aβo produce an increase of SWA (0.5–5 Hz) and low-beta (16–20 Hz) activity during wakefulness at the level of the frontal EEG electrode 24 h after the last day of injection. Moreover, frontal theta (5–9 Hz) and alpha (9–12 Hz) activities during SWS were reduced by soluble Aβo, which was accompanied by a decreased theta activity/SWA ratio during wake and SWS. These changes were not observed before 6 days of administration and occurred together with a decrease in neuron count in the DG. These findings reveal a specific EEG signature of soluble Aβo in the hippocampus and could serve the development of biomarkers specifically for Aβ pathology.

The preservation of wake/sleep amount and architecture following soluble Aβo administration to the hippocampus of rats is in agreement with a previous human study showing no change in sleep quantity before amyloid deposition in the preclinical stage of AD [19]. Our results are also in accordance with a previous study showing no alteration of time spent in wakefulness, SWS, and PS in young APdE9 mice, before the formation of amyloid plaques [22], as well as with findings in TgF344-AD rats [21]. These results align with the role of the hippocampus in generating rhythms of synchronized neuronal activity during wakefulness and sleep (e.g., theta rhythm, sharp wave ripples) [29, 52, 53] and the absence of clear implication of this brain structure in the control of wake/sleep amount and alternation. Indeed, the localized (restricted) distribution of Aβo to the hippocampus (and around the DG) in the current model [6, 28, 35] was not expected to substantially impact the time spent in wake and sleep states. Nevertheless, changes in time spent awake and asleep have been reported in other transgenic AD mouse models with some level of hippocampal Aβ pathology, which includes sleep disturbances before amyloid plaque deposition [20, 25]. Since soluble Aβo accumulation and plaque deposition are not restricted to the hippocampus in these transgenic models, the impact on sleep architecture might originate from the brain regions different from the hippocampus. Given that Aβo were shown to have prion-like properties [54], and considering that the hippocampus has afferent and efferent connections with sleep-regulatory regions (e.g., thalamus, hypothalamus, raphe, locus coeruleus), it would be interesting to investigate the delayed/long-term effects of hippocampal Aβo injections on sleep architecture together with assessing Aβ pathology in key sleep-regulatory regions. This could include expanding intra-hippocampal injections beyond 6 consecutive days. Overall, our findings suggest that alterations in wake/sleep amount and distribution are not a defining feature of the onset of Aβ pathology, at least when considering only the hippocampus.

EEG activity during wakefulness and sleep states was also reported to be modified in AD transgenic models and patients [20]. In general, AD patients show an activity shift towards slower frequencies of the EEG with increased SWA and decreased alpha power [55, 56]. These observations are reminiscent of the findings in the present rat AD model. In healthy individuals, slow-frequency EEG activity is lowest during wakefulness [57] and is considered a reliable EEG index of homeostatic sleep pressure when measured during SWS [58, 59]. Accordingly, an elevated SWA during wakefulness could be considered a sign of impaired wake quality. Our findings suggest that soluble Aβo in the hippocampus are contributing to this change in wake quality. In the present dataset, hippocampal administration of soluble Aβo did not impact theta activity during wake and PS. This may be surprising given the well-recognized implication of the hippocampus in the generation of the theta rhythm [29, 52, 53]. However, medial septum projections to hippocampal CA1 and CA3 regions are also importantly involved in theta generation [29, 53] and could compensate for the Aβo-driven alterations in hippocampal function in the present study.

SWS is considered to be the most restorative sleep stage, and the dissipation in the course of the sleep episode of SWA during SWS is known as a marker of homeostatic recovery during sleep [48–50, 60]. On the one hand, the reduction of theta and alpha activities during SWS that we found after the administration of soluble Aβo to the hippocampus represents a specific modification in the spectral signature of SWS and may suggest an alteration in the quality of this state. This is reminiscent of the worse sleep quality reported in preclinical AD [19]. Similarly, the lower theta activity/SWA ratio during both wakefulness and SWS implies a change in the relationship between synchronized brain activity at different scales, which is known to contribute to cognitive function [61], and could reveal as a distinctive biomarker of hippocampal Aβo. On the other hand, the preserved 24-h dynamics of SWA that we report under Aβo injections points towards an intact sleep homeostasis. An evaluation of the response to sleep loss could refine conclusions regarding the potential impact of soluble Aβo on homeostatic sleep regulation.

Interestingly, a specific signature of Aβo delivered to the dorsal hippocampus was found for the frontal cortex only, which corresponds to the motor cortex with the selected coordinates, but not for the central electrode located above the retrosplenial cortex. It is expected that Aβo localized to the hippocampus impacts neuronal activity via binding to various membrane proteins including receptors and synaptic components [62], which will disrupt outputs of the hippocampus to different cortical regions. Given the connectivity between the hippocampus and the retrosplenial cortex [63, 64], the absence of EEG alterations at the central site is surprising and could be due to injections targeting the dorsal hippocampus but not the anatomically closer ventral hippocampus or to the observation that projecting neurons from the hippocampus to the retrosplenial cortex are GABAergic [63]. In contrast, it is likely that specific alterations in fronto-hippocampal circuits are contributing to the significant effect of Aβo on EEG activity measured at the level of the motor cortex. This finding notably aligns with the findings in AD patients with regard to SWA [65]. Of importance is that a certain threshold of Aβ accumulation and a beginning of cell death in the DG appear necessary for EEG changes since these were observed only 24 h following the last injection on day 6, but not earlier. In addition, the magnitude of the EEG activity changes after 6 days remains relatively modest (3–5%) in comparison with the observations in genetic models of AD (10–30%) [25, 41], for which genetic manipulations are in place during neurodevelopment. Nevertheless, the unique EEG spectral signature of hippocampal soluble Aβo obtained here using electrodes at the surface of the cerebral cortex shows a translational potential as a biomarker of preclinical and/or prodromal stages of AD, when soluble Aβo begin to accumulate before amyloid plaque deposition, tau pathology, brain structure alterations, and cognitive deficits emerge.

### Limitations

Only male rats were used in the present study because the validation of the model was only conducted in males [6, 28, 30, 35], whereas women have a higher risk of developing AD [1, 66]. Wake/sleep disturbances induced by hippocampal Aβo are likely to differ between females and males, especially given the sex differences already present before pathology [67], and further investigation will need to address this question. Another potential limitation of the current research concerns the small area of the hippocampus affected in the chosen model [6, 28, 35], which is not entirely representative of the development of AD and is anticipated to explain the lack of effect on wake/sleep amount and alternation. A second aspect of our experimental design (and constraints) to consider is the use of only one concentration of Aβo. Although justified by the previous publications characterizing the model [6, 28, 30, 35] and lower (thus likely more physiologically/pathologically relevant) than other dosages [68–70], the use of solely one concentration limits the interrogation of dose-dependent effects on wake/sleep variables and the detection of biomarkers associated with a minimal amount of Aβo. In addition, the absence of EEG data recorded later in time after injections (e.g., 3 or 5 days after the end of injections) prevented us from assessing whether the effects of Aβo on EEG activity during wakefulness and SWS are reversible or long-lasting. A last limitation relates to the small number of animals in the presented dataset. Although comparable to previous research [71, 72], a higher number of animals could have allowed identifying additional alterations in wake/sleep phenotypes.

## Conclusions

There is currently no treatment to prevent or cure AD, the most common cause of dementia in the elderly [1]. To achieve this goal, biological markers are needed to detect the disease as early as possible, during the prodromal stages, in order to offer a window to therapeutic interventions before neurodegeneration produces irreversible brain damage and severe cognitive deficits. Unfortunately, reliable biomarkers are still urgently needed [1, 73]. Here, we have found that hippocampal Aβo induced significant alterations of SWA, theta, alpha, and low-beta activities during wakefulness and SWS for the frontal cortex, which could reveal a specific biomarker of early AD. EEG monitoring in humans can be used as a non-invasive and cost-effective diagnostic tool [74, 75], and the present results could not only guide the development of screening tools to detect preclinical AD more efficiently, but also be used as an outcome measure for new AD therapies tested in clinical trials.

### Supplementary Information

**Additional file 1: Table S1.** Results of t-tests comparing BL and INJ for the activity of each frequency band for the central and frontal electrodes in Aβscr et Aβo groups. **Table S2.** Results of one-way ANOVAs comparing BL, D2 and D5 for the activity of each frequency band for the central and frontal electrodes in Aβscr et Aβo groups. **Fig. S1.** Additional variables related to sleep architecture for Aβscr- and Aβo-injected rats. **A** Percent time spent in wake, SWS and PS during the 12 h light (upper panel) and 12 h dark (lower panel) periods of BL and INJ days in Aβscr and Aβo groups. **B** Number of 4-s episodes of wakefulness and SWS. **C** Number of 8- and 16-min episodes of wake, and of 60-s episodes of SWS and PS in Aβscr and Aβo groups during BL and INJ. Aβscr *n* = 11, and Aβo *n* = 9. **Fig. S2.** Additional data concerning wake, SWS and PS spectral activity during BL and INJ in Aβo and Aβscr rats. **A** Spectral activity for each frequency band for the central and frontal electrodes. SWA: 0.5–5 Hz; theta: 5–9 Hz; alpha: 9–12 Hz; sigma: 12–16 Hz; beta1: 16–20 Hz; beta2: 20–30 Hz; gamma: 30–55 Hz. **B** Ratio of theta activity on gamma activity for the central (Aβo and Aβscr rats) and frontal (Aβscr rats) electrodes. **C** Ratio of theta activity on SWA for the central (Aβo and Aβscr rats) and frontal (Aβscr rats) electrodes. Aβscr *n* = 9, and Aβo *n* = 6. **Fig. S3.** Wake, SWS and PS spectral activity for each frequency band for the central and frontal electrodes at BL and at D2 and D5 in Aβscr-injected rats (*n* = 8). SWA: 0.5–5 Hz; theta: 5–9 Hz; alpha: 9–12 Hz; sigma: 12–16 Hz; beta1: 16–20 Hz; beta2: 20–30 Hz; gamma: 30–55 Hz.

## Data Availability

The datasets used and analyzed in the current study are available from the corresponding authors upon reasonable request.

## Abbreviations

Aβo: Amyloid-beta oligomers
Aβscr: Amyloid-beta scrambled
AD: Alzheimer’s disease
aMCI: Amnestic mild cognitive impairment
ANOVA: Analysis of variance
APdE9: APPswe/PS1dE9
APP: Amyloid precursor protein
BL: Baseline
D2: Day 2
D5: Day 5
DMSO: Dimethylsulfoxide
EEG: Electroencephalographic
EMG: Electromyographic
HIFP: Hexafluoroisopropanol
IHC: Immunohistochemistry
INJ: Twenty-four hours after the last injection
PS: Paradoxical sleep
SWA: Slow-wave activity
SWS: Slow-wave sleep
