# Supplementary material for: Hippocampal injections of soluble amyloid-beta oligomers alter electroencephalographic activity during wake and slow-wave sleep in rats
Source: Alzheimers Res Ther. 2023 Oct 13;15:174. doi: 10.1186/s13195-023-01316-4 (PMC10571363; doi:10.1186/s13195-023-01316-4)
Supplement: Supplementary file 1 — Additional file 1: Table S1. Results of t-tests comparing BL and INJ for the activity of each frequency band for the central and frontal electrodes in Aβscr et Aβo groups. Table S2. Results of one-way ANOVAs comparing BL, D2 and D5 for the activity of each frequency band for the central and frontal electrodes in Aβscr et Aβo groups. Fig. S1. Additional variables related to sleep architecture for Aβscr- and Aβo-injected rats. A Percent time spent in wake, SWS and PS during the 12 h light (upper panel) and 12 h dark (lower panel) periods of BL and INJ days in Aβscr and Aβo groups. B Number of 4-s episodes of wakefulness and SWS. C Number of 8- and 16-min episodes of wake, and of 60-s episodes of SWS and PS in Aβscr and Aβo groups during BL and INJ. Aβscr n = 11, and Aβo n = 9. Fig. S2. Additional data concerning wake, SWS and PS spectral activity during BL and INJ in Aβo and Aβscr rats. A Spectral activity for each frequency band for the central and frontal electrodes. SWA: 0.5–5 Hz; theta: 5–9 Hz; alpha: 9–12 Hz; sigma: 12–16 Hz; beta1: 16–20 Hz; beta2: 20–30 Hz; gamma: 30–55 Hz. B Ratio of theta activity on gamma activity for the central (Aβo and Aβscr rats) and frontal (Aβscr rats) electrodes. C Ratio of theta activity on SWA for the central (Aβo and Aβscr rats) and frontal (Aβscr rats) electrodes. Aβscr n = 9, and Aβo n = 6. Fig. S3. Wake, SWS and PS spectral activity for each frequency band for the central and frontal electrodes at BL and at D2 and D5 in Aβscr-injected rats (n = 8). SWA: 0.5–5 Hz; theta: 5–9 Hz; alpha: 9–12 Hz; sigma: 12–16 Hz; beta1: 16–20 Hz; beta2: 20–30 Hz; gamma: 30–55 Hz. [file 13195_2023_1316_MOESM1_ESM.pdf]

Additional file supporting:

Hippocampal injections of soluble amyloid-beta oligomers alter electroencephalographic activity during wake and slow wave sleep in rats

Audrey Hector, Chloé Provost, Benoît Delignat-Lavaud, Khadija Bouamira,

Chahinez-Anissa Menaouar, Valérie Mongrain, Jonathan Brouillette

Published in: *Alzheimer's Research & Therapy*

This file contains:

- Supplementary Tables S1 and S2
- Supplementary Fig. S1 to S3

Supplementary Tables

Table S1 Results of t-tests comparing BL and INJ for the activity of each frequency band for the central and frontal electrodes in Aβscr et Aβo groups.

**Aβscr**		Central		Frontal		**Aβo**		Central		Frontal	
		t	*p*	t	*p*			t	*p*	t	*p*
SWA	Wake	0.47	0.65	0.89	0.40	SWA	Wake	0.71	0.51	2.71	0.04
	SWS	0.87	0.41	0.98	0.36		SWS	0.37	0.73	0.47	0.66
	PS	0.83	0.43	1.24	0.25		PS	0.06	0.95	1.18	0.29
Theta	Wake	1.30	0.23	0.93	0.38	Theta	Wake	0.10	0.93	0.80	0.46
	SWS	0.11	0.92	0.45	0.67		SWS	1.51	0.19	5.66	<0.01
	PS	0.81	0.44	0.20	0.85		PS	0.78	0.47	0.90	0.41
Alpha	Wake	0.70	0.50	1.15	0.28	Alpha	Wake	0.75	0.48	1.87	0.12
	SWS	0.06	0.95	0.98	0.36		SWS	1.25	0.27	3.15	0.03
	PS	0.89	0.40	0.82	0.44		PS	0.92	0.40	0.00	0.99
Sigma	Wake	1.23	0.26	0.29	0.78	Sigma	Wake	0.51	0.63	1.73	0.14
	SWS	0.33	0.75	2.18	0.06		SWS	2.19	0.08	2.22	0.08
	PS	0.06	0.96	1.12	0.30		PS	0.71	0.51	0.32	0.76
Beta1	Wake	1.27	0.24	1.36	0.21	Beta1	Wake	0.66	0.54	3.17	0.02
	SWS	0.78	0.46	1.02	0.34		SWS	1.90	0.12	1.93	0.11
	PS	0.46	0.66	1.13	0.29		PS	0.61	0.57	0.14	0.89
Beta2	Wake	1.22	0.26	1.15	0.29	Beta2	Wake	0.53	0.62	2.00	0.10
	SWS	0.76	0.47	1.43	0.23		SWS	1.27	0.26	1.27	0.26
	PS	0.43	0.68	0.01	0.99		PS	0.43	0.68	0.12	0.91
Gamma	Wake	0.72	0.49	0.70	0.50	Gamma	Wake	0.99	0.37	0.16	0.88
	SWS	0.78	0.46	0.63	0.54		SWS	0.78	0.47	0.48	0.65
	PS	0.33	0.75	1.08	0.31		PS	0.37	0.37	0.90	0.41

Table S2 Results of one-way ANOVAs comparing BL, D2 and D5 for the activity of each frequency band for the central and frontal electrodes in Aβscr et Aβo groups.

**Aβscr**		Central		Frontal		**Aβo**		Central		Frontal	
		$F_{2,14}$	p	$F_{2,14}$	p			$F_{2,8}$	p	$F_{2,8}$	p
SWA	Wake	2.12	0.19	0.91	0.42	SWA	Wake	0.62	0.49	1.81	0.25
	SWS	1.50	0.26	1.07	0.37		SWS	0.41	0.60	3.32	0.12
	PS	0.81	0.41	1.67	0.23		PS	0.56	0.52	1.84	0.22
Theta	Wake	0.28	0.67	1.52	0.26	Theta	Wake	1.00	0.38	0.83	0.47
	SWS	2.46	0.16	4.02	0.07		SWS	0.43	0.66	0.69	0.47
	PS	3.50	0.10	0.57	0.51		PS	1.12	0.37	0.50	0.53
Alpha	Wake	0.93	0.36	0.16	0.85	Alpha	Wake	1.29	0.32	2.66	0.15
	SWS	3.46	0.10	0.90	0.42		SWS	0.86	0.46	1.88	0.22
	PS	2.31	0.17	1.74	0.21		PS	2.77	0.12	0.06	0.85
Sigma	Wake	0.40	0.57	0.06	0.91	Sigma	Wake	0.73	0.49	2.15	0.16
	SWS	3.35	0.10	1.39	0.27		SWS	0.65	0.52	0.22	0.81
	PS	2.94	0.13	0.55	0.55		PS	1.80	0.24	0.52	0.57
Beta 1	Wake	0.17	0.74	1.02	0.37	Beta 1	Wake	0.73	0.46	4.77	0.06
	SWS	1.84	0.21	2.58	0.10		SWS	1.28	0.33	1.33	0.32
	PS	2.55	0.15	0.80	0.44		PS	1.27	0.33	0.40	0.60
Beta 2	Wake	0.09	0.83	0.14	0.80	Beta 2	Wake	0.82	0.42	0.69	0.52
	SWS	0.46	0.55	0.52	0.60		SWS	1.17	0.36	0.27	0.76
	PS	2.35	0.17	0.66	0.48		PS	0.92	0.41	0.31	0.68
Gamma	Wake	1.80	0.21	0.23	0.70	Gamma	Wake	1.26	0.33	0.93	0.41
	SWS	0.07	0.83	0.56	0.53		SWS	1.73	0.25	1.16	0.34
	PS	3.23	0.10	0.10	0.86		PS	1.18	0.35	0.71	0.46

Supplementary Figures

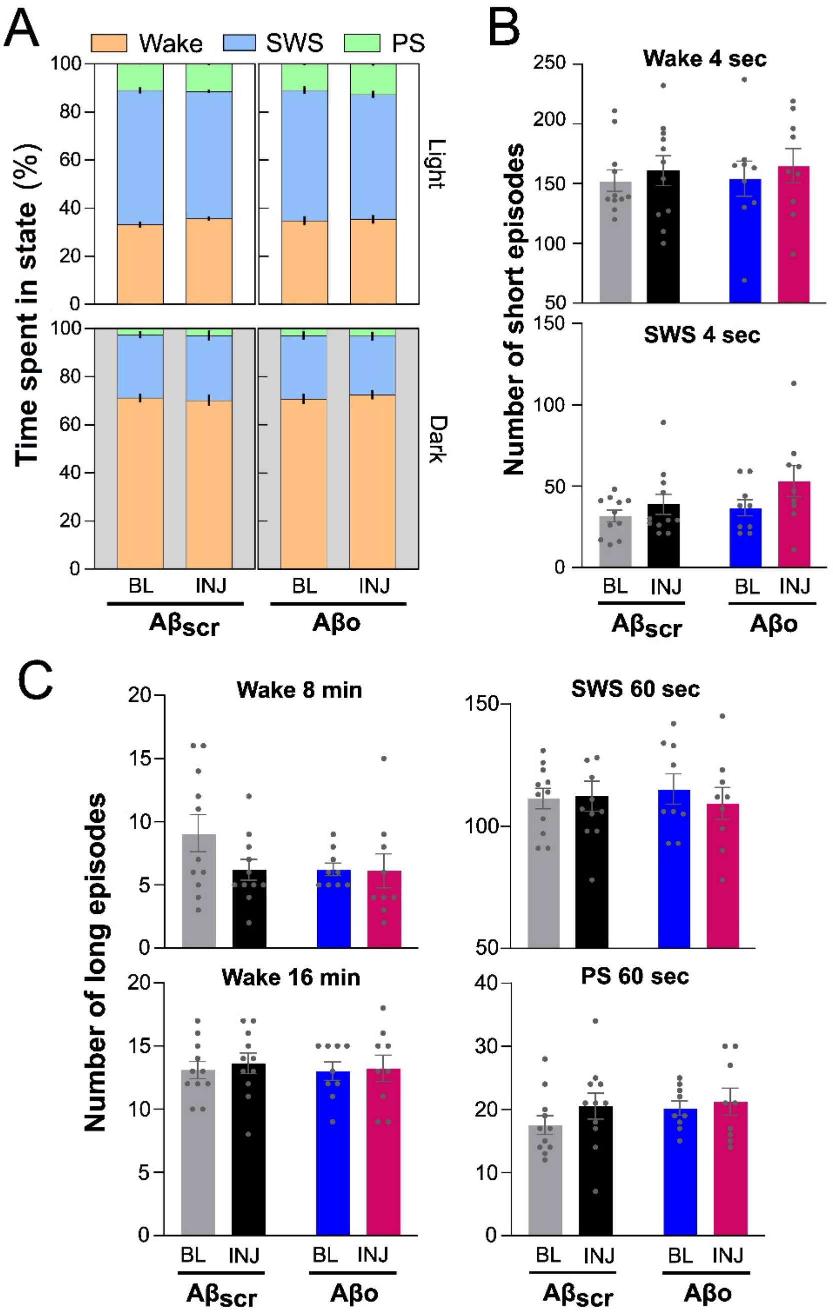

Fig. S1 Additional variables related to sleep architecture for Aβscr- and Aβo-injected rats. **A** Percent time spent in wake, SWS and PS during the 12 h light (upper panel) and 12 h dark (lower panel) periods of BL and INJ days in Aβscr and Aβo groups. **B** Number of 4-sec episodes of wakefulness and SWS. **C** Number of 8- and 16-min episodes of wake, and of 60-sec episodes of SWS and PS in Aβscr and Aβo groups during BL and INJ. Aβscr $n = 11$, and Aβo $n = 9$.

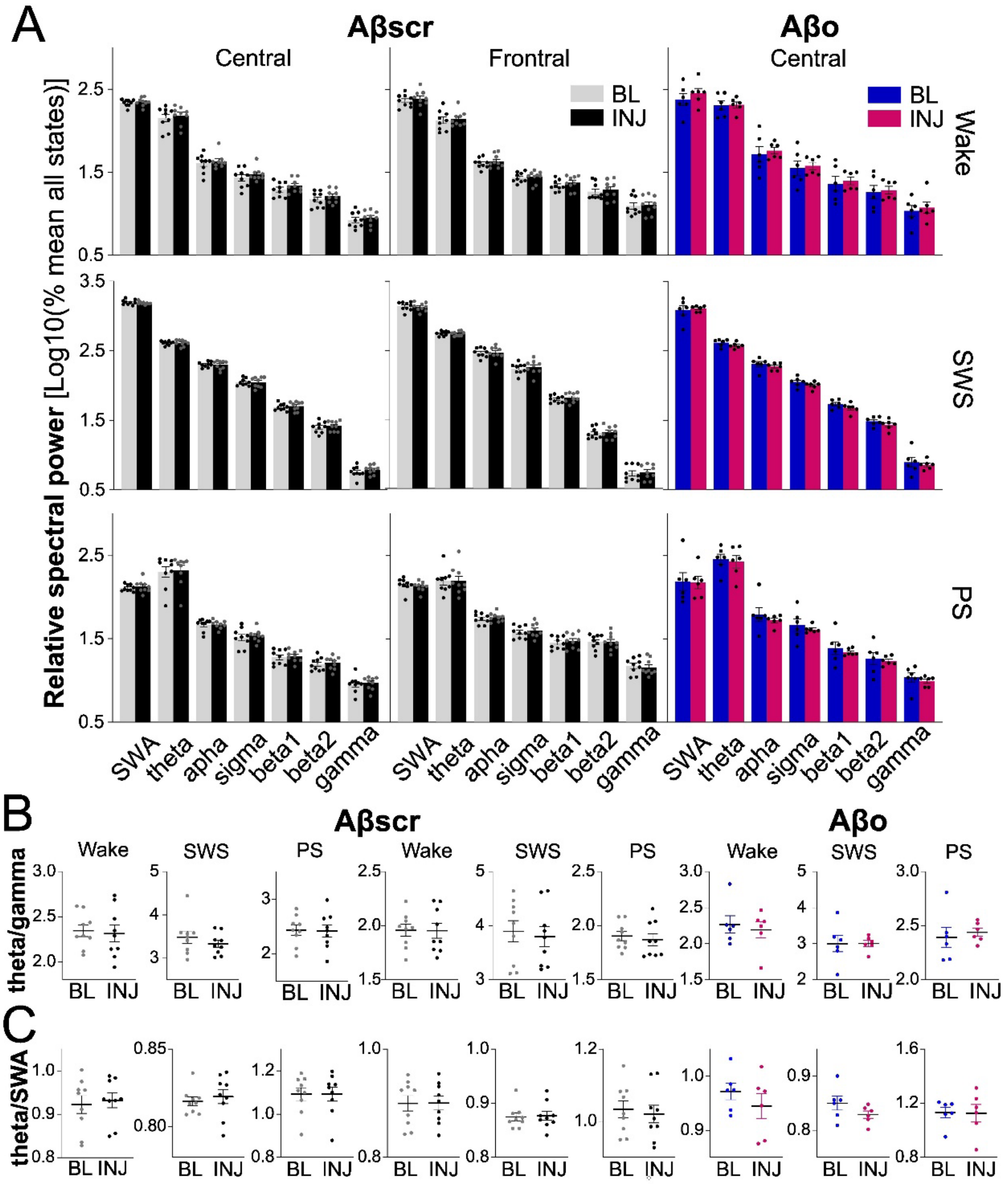

Fig. S2 Additional data concerning wake, SWS and PS spectral activity during BL and INJ in Aβo and Aβscr rats. **A** Spectral activity for each frequency band for the central and frontal electrodes. SWA: 0.5-5 Hz; theta: 5-9 Hz; alpha: 9-12 Hz; sigma: 12-16 Hz; beta1: 16-20 Hz; beta2: 20-30 Hz; gamma: 30-55 Hz. **B** Ratio of theta activity on gamma activity for the central (Aβo and Aβscr rats) and frontal (Aβscr rats) electrodes. **C** Ratio of theta activity on SWA for the central (Aβo and Aβscr rats) and frontal (Aβscr rats) electrodes. Aβscr $n = 9$, and Aβo $n = 6$.

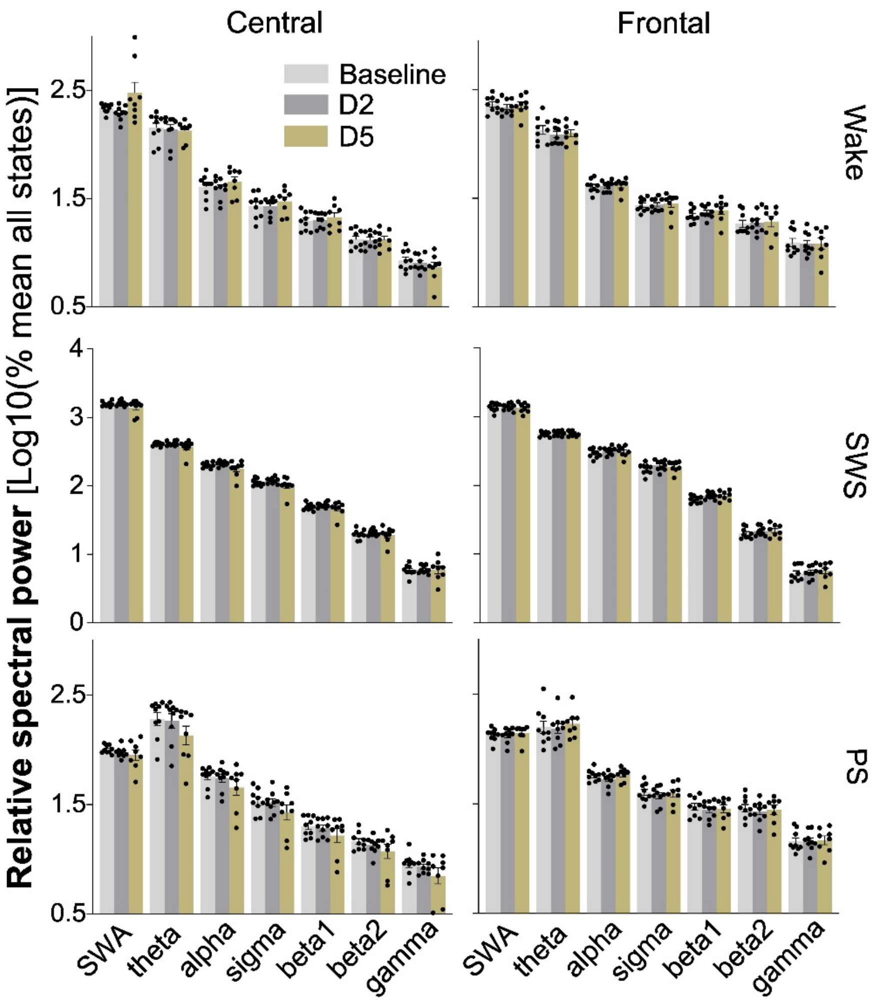

Fig. S3 Wake, SWS and PS spectral activity for each frequency band for the central and frontal electrodes at BL and at D2 and D5 in Aβscr-injected rats ($n = 8$). SWA: 0.5-5 Hz; theta: 5-9 Hz; alpha: 9-12 Hz; sigma: 12-16 Hz; beta1: 16-20 Hz; beta2: 20-30 Hz; gamma: 30-55 Hz.
